# An ERP Investigation of L2–L1 Translation Priming in Adult Learners

**DOI:** 10.3389/fpsyg.2018.00986

**Published:** 2018-06-19

**Authors:** Gabriela Meade, Katherine J. Midgley, Phillip J. Holcomb

**Affiliations:** ^1^Joint Doctoral Program in Language and Communicative Disorders, San Diego State University and University of California, San Diego, San Diego, CA, United States; ^2^Department of Psychology, San Diego State University, San Diego, CA, United States

**Keywords:** translation priming, second language acquisition, word learning, lexical mediation, semantic mediation, bilingualism, ERPs

## Abstract

A longstanding debate centers around how beginning adult bilinguals process words in their second language (L2). Do they access the meaning of the L2 words directly or do they first activate the native language (L1) translation equivalents in order to access meaning? To address this question, we used ERPs to investigate how newly learned L2 words influence processing of their L1 translation equivalents. We taught participants the meanings of 80 novel L2 (pseudo)words by presenting them with pictures of familiar objects. After 3 days of learning, participants were tested in a backward translation priming paradigm with a short (140 ms) stimulus onset asynchrony. L1 targets preceded by their L2 translations elicited faster responses and smaller amplitude negativities than the same L1 targets preceded by unrelated L2 words. The bulk of the ERP translation priming effect occurred within the N400 window (350–550 ms), suggesting that the new L2 words were automatically activating their semantic representations. A weaker priming effect in the preceding window (200–350 ms) was found at anterior sites, providing some evidence that the forms of the L1 translation equivalents had also been activated. These results have implications for models of L2 processing at the earliest stages of learning.

## Introduction

Adult learners of a second language (L2) already have an established system of linguistic and conceptual knowledge in their native language (L1). How L2 words are integrated into that system as they are learned continues to be debated (e.g., Jiang, 2000; Jiang and Forster, 2001; Brysbaert and Duyck, 2010; Grainger et al., 2010; Kroll et al., 2010; Ma et al., 2017; Meade and Dijkstra, 2017). The debate is motivated by leading theories of sequential bilingualism, including the Revised Hierarchical Model (RHM; e.g., Kroll and Stewart, 1994; Kroll et al., 2010) and the Developmental Bilingual Interactive-Activation Model (BIA-d; Grainger et al., 2010), which posit that L2 processing differs as a function of proficiency. At high levels of proficiency, bilinguals are thought to process L1 and L2 words similarly, with direct connections between lexical representations in both languages and a shared semantic store. At earlier stages of proficiency, these models posit that new L2 words are primarily processed via their L1 translation equivalents (i.e., through lexical mediation). However, recent evidence has begun to contradict the latter, suggesting that more direct access to semantics might be established even in low proficiency bilinguals (see, e.g., Duyck and Brysbaert, 2004; Ma et al., 2017; Meade and Dijkstra, 2017). To further investigate whether new L2 words initially activate their L1 translation equivalents or whether they go directly to semantics, we taught participants a set of 80 L2 words and tested them in a backward (L2–L1) translation ERP priming paradigm with a short stimulus onset asynchrony (SOA).

Evidence for forward and backward translation priming in proficient bilinguals comes from a number of behavioral studies using a primed lexical decision task. L1 target words are classified as real words faster when they are preceded by their L2 translation equivalents than when they are preceded by unrelated L2 words, and the same is true for L2 targets preceded by L1 primes (see, e.g., Duñabeitia et al., 2010b; Wen and van Heuven, 2017, for a meta-analysis). Theoretically, this translation priming could be either due to spreading of activation from the prime word to the form representation of the target word or due to spreading of activation to a shared semantic representation.

Several lines of research converge to suggest that in proficient bilinguals the effect is largely due to facilitated semantic processing. ERPs have played a critical role in pinpointing the semantic nature of translation priming in proficient bilinguals by lending insight into how the priming effect develops over time. Broadly speaking, semantic priming is associated with the N400 and form priming is associated with earlier components, including the P200 and the N250 (e.g., Grainger and Holcomb, 2009; Guo et al., 2012).^1^ In a go/no-go lexical decision study with late proficient Russian–English bilinguals, Geyer et al. (2011) time-locked ERPs to L1 and L2 words preceded by the identical word in the same language, by the translation equivalent, or by an unrelated word. In general, words primed by related items elicited smaller negativities than words primed by unrelated items. The identity priming effect in both L1 and L2 began in the earliest window measured (150–300 ms) while the forward and backward translation priming effects were only observed within the N400 window (300–500 ms). The authors interpreted this pattern to suggest that both form and meaning were primed in the identity conditions, whereas only meaning was primed in the translation conditions (see also, Phillips et al., 2006). Duñabeitia et al. (2010a) reported a similar pattern with balanced Basque–Spanish bilinguals in a go/no-go semantic categorization masked priming paradigm; identity priming effects were found within both the N250 and N400 windows, but translation priming effects were restricted to the N400 window. Thus, the timing of the translation priming effect in proficient bilinguals is more consistent with facilitated semantic processing than with facilitated processing of the form of the translation equivalent.

The translation recognition paradigm is another approach to probing the mechanisms that underlie translation priming and, by extension, L2 word processing. In this paradigm, participants see pairs of words and decide whether the two words are correct translations of one another. On critical trials, the L1 target is not the correct translation (e.g., *ajo*) of the L2 prime (e.g., *garlic* for Spanish–English bilinguals), but is related to it either in form (e.g., *ojo* is a form neighbor of *ajo*, but has the semantically unrelated meaning ‘eye’) or meaning (e.g., cebolla means ‘onion’; e.g., Talamas et al., 1999). In proficient bilinguals, both form and semantic distractors produce behavioral interference effects (i.e., slower and less accurate responses compared to unrelated incorrect translations; e.g., Altarriba and Mathis, 1997; Ferré et al., 2006; Moldovan et al., 2016). This suggests that both the meanings and the form of the translation equivalents are activated and make it more difficult to reject the distractors as incorrect translations.

Nevertheless, in proficient bilinguals the behavioral interference effect tends to be larger for semantic distractors than for form distractors, reinforcing that meaning plays a major role in processing of L2 words (e.g., Talamas et al., 1999; Ferré et al., 2006). In an ERP translation recognition task, Guo et al. (2012) also demonstrated that the semantic pathway is more automatic than the lexical pathway in proficient bilinguals. At a 750 ms SOA, form distractors elicited larger amplitude P200s than unrelated targets and semantic distractors elicited smaller amplitude N400s than unrelated targets. Consistent with behavioral results, this pattern suggests that L2 primes were activating both their meanings and the forms of their translation equivalents. However, at a 300 ms SOA, the effect for form distractors within the P200 window disappeared. This prompted the authors to suggest that semantic representations were activated before the L1 translation equivalents (see also, Moldovan et al., 2016). Guo et al.’s (2012) electrophysiological data and SOA manipulations provided detailed time-course information that supports semantics as a primary source of translation priming for proficient bilinguals. This conclusion is consistent with the RHM and the BIA-d in that both models posit direct semantic access for L2 words in proficient bilinguals.

The question that remains unanswered is whether facilitated semantic processing also underlies translation priming in less proficient bilinguals, for whom these theoretical models posit a different lexical architecture. Both the RHM and the BIA-d postulate that L2 words are only directly connected to their L1 translation equivalents in less proficient bilinguals. Therefore, backward translation priming should be lexically mediated, with pre-activation of the form of the L1 translation equivalent as the primary catalyst of the priming effect. Previous empirical studies with less proficient bilinguals have yielded mixed results. For one, it is not clear whether backward translation priming even occurs in the lexical decision task in these less proficient bilinguals (e.g., Jiang and Forster, 2001; Duyck and Warlop, 2009; Dimitropoulou et al., 2011a,b; Witzel and Forster, 2012). For another, the approaches described above to dissociate between the contributions of the semantic representation versus the L1 translation equivalent have been inconclusive.

Early behavioral evidence from translation recognition paradigms was consistent with processing of L2 words via lexical mediation, as proposed in the RHM and BIA-d. The finding of an interference effect for form distractors (e.g., *ojo* instead of *ajo*) was interpreted to suggest that lower proficiency bilinguals were relying on activation of the L1 translation equivalent to process L2 words (e.g., Talamas et al., 1999; Ferré et al., 2006). This argument was especially convincing given the absence of the analogous effect for semantic distractors in the same participants (i.e., no significant differences in response times between semantic distractors and unrelated targets). In other words, it appeared that the form of the L1 translation equivalent was being activated but the semantic representation was not, perfectly in line with model predictions.

Reports of semantic interference in translation recognition tasks have since challenged the original null semantic finding, indicating that the meanings of the L2 primes can be activated in low proficiency bilinguals under certain conditions (e.g., Sunderman and Kroll, 2006). It is difficult to determine on the basis of these behavioral data alone whether the semantic interference – when it is present – is driven by direct activation of the meaning or indirect semantic activation via the L1 translation equivalent. This is especially true given that these studies used a relatively long 500 ms SOA, which presumably allowed sufficient time for the indirect route. However, recent ERP data from a translation recognition task with lower proficiency bilinguals points to semantics as the primary processing pathway for L2 words (Ma et al., 2017). Similar to proficient bilinguals (Guo et al., 2012), at a 300 ms SOA, there was no effect for form distractors within the P200 window, but there was an effect for semantic distractors within the N400 window. Thus, although translation recognition behavioral data have been inconclusive, recent ERP evidence suggests that L2 comprehension might be semantically mediated, even in unbalanced bilinguals.

Results from standard ERP masked priming paradigms with unbalanced bilinguals also appear more consistent with direct semantic access. For example, Midgley et al. (2009) failed to find evidence of backward translation priming within the N250 window using a 67 ms SOA. The translation priming effect for L1 targets preceded by masked L2 translation primes was restricted to the N400 window, suggesting that the L2 primes were activating their meanings, but not the forms of their L1 translation equivalents. However, in a subsequent masked priming study with slightly more proficient participants and a longer (120 ms) SOA, Schoonbaert et al. (2011) found a widespread N250 priming effect for L1 targets preceded by L2 primes (i.e., smaller negativities for L1 targets in translation pairs compared to those in unrelated pairs). Note that, theoretically, the backward translation N250 priming effect should decrease as proficiency increases and reliance on the L1 translation equivalent diminishes, which is opposite the pattern found across these studies. Instead, the authors suggest that the longer SOA in the study by Schoonbaert et al. (2011) allowed participants to process the L2 primes enough to activate L1 translation equivalents at the form level. Given that backward translation N400 priming effects were robust even at the shorter SOA, these studies seem to suggest that meaning is the primary processing pathway for L2 words, even before high levels of proficiency are achieved.

In interpreting these studies, it is important to keep in mind that the bilingual participants, though unbalanced, had relatively high levels of L2 proficiency. For example, although Midgley et al. (2009) categorized their participants as second language learners, the participants’ average self-ratings of L2 language skills were about 4 on a Likert scale from 1 (unable) to 7 (expert). Accurately quantifying the proficiency level of bilinguals who have learned in a classroom and/or immersion setting is challenging (e.g., Grosjean, 1998) and can differ depending on the measurement tool (e.g., Gollan et al., 2012). At the same time, the proficiency level at which the theoretical transition from lexical mediation to semantic mediation occurs has yet to be specified. Therefore, it remains possible that these participants had already surpassed the proficiency level at which the transition takes place, which would make the evidence of semantic mediation in relatively low proficiency bilinguals more consistent with the theoretical models.

Testing for translation priming effects in the context of a word learning experiment is one way to circumvent this issue of when the transition from lexical mediation to direct semantic access occurs. In fact, deconstructing translation pathways in participants who begin learning their L2 as part of the experiment would seem to be one of the most rigorous tests of the theoretical models that propose lexical mediation at low levels of proficiency. A handful of such priming studies with learners have been conducted, but have failed to yield conclusive results thus far (e.g., Altarriba and Mathis, 1997; Mestres-Missé et al., 2007; Dobel et al., 2009; Witzel and Forster, 2012; Pu et al., 2016). For example, after teaching English monolinguals a set of 36 Spanish words, Altarriba and Mathis (1997) found behavioral interference effects for both form and semantic distractors in a translation recognition task with a 300 ms SOA. This suggests that both the L1 translation equivalent forms and the meanings of the new L2 words were activated. Emerging ERP evidence supports the claim that L2 words activate both their meanings and the forms of their translation equivalents in learners (e.g., Mestres-Missé et al., 2007; Pu et al., 2016). For example, Pu et al. (2016) taught native English speakers 112 Spanish words through explicit paired associations (e.g., *cama*-*bed*) and tested them in translation verification task (i.e., are these word pairs correct translations?). They found that targets in translation pairs elicited smaller negativities than targets in unrelated pairs (i.e., priming) beginning between 200 and 300 ms and continuing through the N400 window. The authors interpret the early onset of the priming effect as support for lexically mediated backward translation, but acknowledge that the 800 ms SOA was long enough to allow for strategic activation of the form of the L1 translation. Especially in light of recent findings that the duration of the SOA influences ERP priming patterns in low proficiency bilinguals (Ma et al., 2017), it is important to test whether or not these priming patterns hold at a short SOA.

In summary, there is robust evidence for N400 effects in translation priming studies, which supports semantic mediation among bilinguals at all levels of proficiency (e.g., Midgley et al., 2009; Duñabeitia et al., 2010a; Geyer et al., 2011; Schoonbaert et al., 2011; Guo et al., 2012; Pu et al., 2016; Ma et al., 2017). The evidence for earlier, form-based priming effects is comparatively limited and is mostly observed in studies with long SOAs (e.g., Guo et al., 2012; Pu et al., 2016; Ma et al., 2017). However, almost all of these studies have been done with bilinguals who had already achieved some L2 proficiency. To further investigate whether L2 words are processed via lexical mediation at the earliest stages of learning, we taught participants a set of novel L2 words and tested them in a backward priming paradigm with a 140 ms SOA. The L2 words were initially pseudowords that were paired with pictures representing their meanings during the learning phase of the experiment. Following Pu et al. (2016), after learning we recorded EEG as participants saw L2 prime – L1 target pairs and decided whether the two words were correct translations or not. Using a shorter SOA than in the study by Pu et al. (2016) allowed us to minimize overt translation and index the representations that are automatically and rapidly activated during processing of newly learned L2 words. We predicted that L1 targets preceded by their L2 translations would elicit faster responses and smaller amplitude negativities (i.e., priming) than L1 targets preceded by unrelated L2 words. As argued above, the onset of ERP effects is critical for determining whether translation priming is lexically or semantically motivated in these learners. A priming effect solely within the N400 window would be consistent with activation of the semantic representation. Finding an effect before the N400, in time windows that are commonly associated with processing of lexical form (i.e., P200/N250), would suggest that the form of the L1 translation had been activated during processing of the L2 prime. The latter would be consistent with the lexical mediation posited for low proficiency bilinguals in the RHM and BIA-d.

## Materials and Methods

### Participants

Participants included 18 young adults who were right-handed and had normal or corrected-to-normal vision. By self-report, they were not fluent in any language other than English and were not exposed to any language other than English before the age of 6. Participants reported having no history of neurological dysfunction or language disorders, and were not taking any medications that would affect brain function. Data from these same participants in other tasks have been reported elsewhere (Meade et al., 2018). In addition to the three participants excluded from the original report, data from two additional participants were excluded here. One participant was excluded for high artifact rejection due to blinks in this task (>30% of trials) and the other was the participant with the lowest overall accuracy in the priming task that could be rejected to maintain the counterbalancing described below.^2^

### Stimuli

Stimuli included 86 L2 words (80 critical items and 6 practice items) that were drawn from the ARC Nonword Database (Rastle et al., 2002) and chosen to be orthotactically and phonotactically legal in English (e.g., *grif*, *labe*, *slont*, and *plurd*). All of the L2 words were four to five letters in length; more characteristics of these L2 words and their L1 translations can be found in **Table 1**. During the learning exercises, the L2 words were paired with pictures depicting familiar objects. All of the pictures had naming agreement at or above 85% in previous norming studies (mean = 97%; Bates et al., 2003). Form overlap between the L2 words and their L1 translation equivalents was minimized. The average Levenshtein distance (i.e., number of insertions, deletions, and substitutions) between the L2 words and their L1 translations was 5.12 (*SD*: 1.20). A full description of the L2 words can be found in Meade et al. (2018).

**Table 1:** Stimulus characteristics [mean (SD)].

	Length	N	Frequency	Concreteness
L2 words	4.50 (0.50)	4.35 (4.42)	–	–
L1 translations	5.39 (1.64)	4.98 (5.63)	79.69 (252.81)	4.54 (0.33)


*N = the number of orthographic neighbors in English. N and frequency were extracted from the MCWord database (Medler and Binder, 2005). Concreteness ratings (on a scale from 1 = abstract to 5 = concrete) are from Brysbaert et al. (2014).*

### Procedure

Participants were instructed that they would be learning words from a new language. In order to reinforce that these were words from another language, the experiment began with a language decision ERP pretest (i.e., press one button for English words and another button for words from another language; see Meade et al., 2018). Learning exercises were then administered over three consecutive days beginning the day of the pretest. Each word was presented a total of 12 times during the learning phase, either in the context of a two-alternative forced-choice (2AFC) task or a typing task (see **Table 2**). In the 2AFC task, a picture was presented with two L2 words and participants had to choose which of the L2 words matched the picture. Participants received feedback after each trial in which the picture was displayed together with the correct L2 word. In the typing task, they saw the picture and had to type the corresponding L2 word. If they typed the correct word, they moved on to the subsequent trial. If they typed the incorrect word, the correct word was displayed and they were then asked to type the correct word. On Day 1 of training, they had the first and last letters of the word as a cue in the typing task, but by the last session they had no cues (see **Table 2**). By the last learning session, mean accuracy was 99% (*SD*: 1.2%) in the 2AFC task and 95% (*SD*: 4.3%) in the typing task, which demonstrates that the participants had successfully learned the words.

**Table 2:** Training overview.

Day 1	Day 2	Day 3
Paired associate/2AFC	2AFC	2AFC
2AFC	Typing^‡^	Typing^†^
Typing^‡^	2AFC	2AFC
	Typing^†^	Typing


*2AFC = Two-alternative forced-choice. ^‡^First and last letters provided as a cue. ^†^First letter provided as a cue. Data from these tasks are available in Meade et al. (2018).*

On the fourth day of the experiment, participants took part in an ERP post-test that included a backward priming paradigm. An L2 prime was presented in lowercase for 140 ms, followed immediately by an L1 target in uppercase that remained on the screen for 500 ms. Participants were asked to decide as quickly as possible whether the two words were correct translations and to press one button if they were and another button if they were not. Response hand was counterbalanced across participants. One thousand ms after the response, a purple fixation cross appeared, during which participants were instructed to blink if needed. After 1500 ms, the purple fixation cross turned white for 900 ms and then a 500 ms blank screen signaled the beginning of the next trial. Before beginning the experiment, there was a practice that included three translation and three unrelated trials, none of which were included in the actual experiment.

Each L2 word was presented twice, followed by the correct L1 translation in one half of the experiment and by an unrelated L1 word in the other half. All participants saw the same list (e.g., *grif-ORANGE* in the first half and *grif-KNIGHT* in the second half). However, the pairings between the words and pictures during the learning phase were systematically controlled across participants such that any given pair was the correct translation for half of the participants and unrelated for the other half (e.g., nine participants learned the L2 word *grif* with a picture of an orange and nine of them learned the L2 word *grif* with a picture of a knight). This design ensured that the same L1 targets occurred in the translation and unrelated conditions.

### EEG Recording and Analysis

EEG was recorded from 29 electrodes in an Electro-Cap using a left mastoid reference. It was amplified with SynAmpsRT amplifiers (Neuroscan-Compumedics) using a band pass of DC to 100 Hz and was sampled continuously at 500 Hz. Off-line, ERPs were time-locked to target onset for each participant and prime condition (translation and unrelated) separately using a 100 ms pre-stimulus baseline and a 15 Hz low-pass filter. A loose electrode placed below the left eye was used in conjunction with recordings from FP1 to detect blink artifacts and another electrode on the outer canthus of the right eye was used to detect horizontal eye movements. Impedances were maintained below 10 kΩ for eye electrodes and below 2.5 kΩ for scalp and reference electrodes. Trials with artifacts during the baseline period or within 1000 ms of target onset were excluded from analyses, as were trials with incorrect responses. In the final analyses, an average of 72 and 76 trials (out of 80) were included in the translation and unrelated conditions, respectively.

**FIGURE 1:**
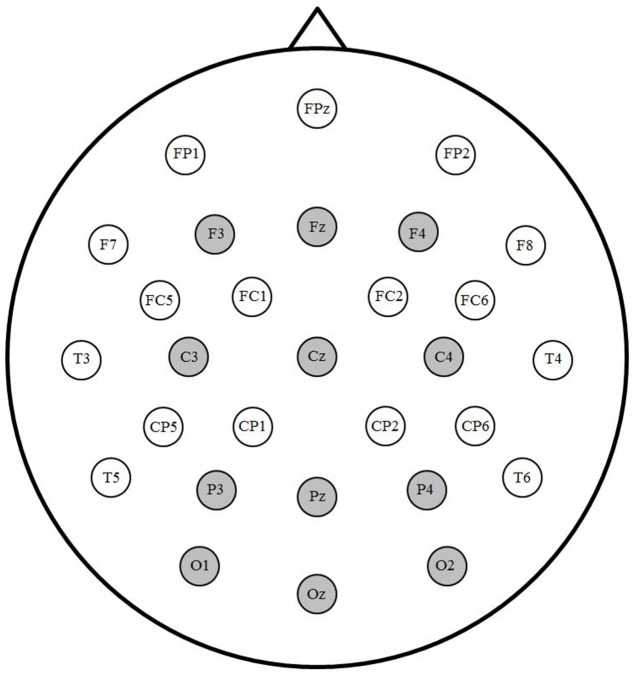
Electrode montage. Sites indicated in gray were included in analyses.

A subset of 12 electrodes was selected for statistical analyses (see **Figure 1**). To test for a translation priming effect, ANOVAs with factors Prime (translation and unrelated), Laterality (left, midline, and right) and Anterior/Posterior (frontal, central, parietal, and occipital) were used on mean amplitude within two successive windows. N400 amplitude was measured between 350 and 550 ms, consistent with previous priming studies (e.g., Grainger et al., 2006; Phillips et al., 2006). Due to the short SOA, processing of the prime and target overlapped in time and the morphology of the waveform differed from standard ERPs to single words. The early window (200–350 ms) was chosen based on visual inspection of the grand averaged waveforms to encompass the negative peak preceding the N400.

## Results

**FIGURE 2:**
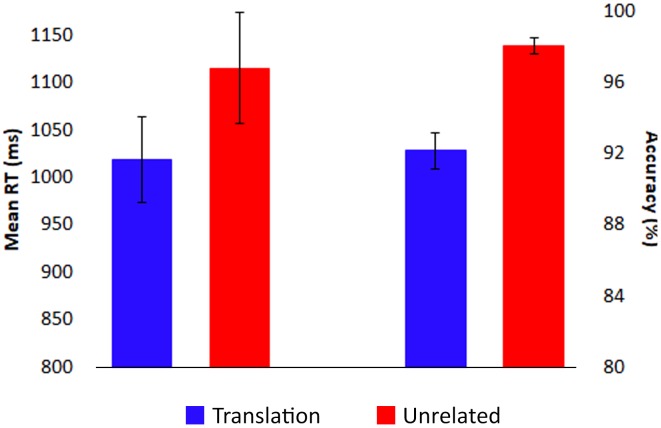
Behavioral results. Responses were faster (left) and less accurate (right) for targets in translation pairs (blue) than for targets in unrelated pairs (red). Bars indicate standard error.

Response times shorter than 200 ms and longer than 2000 ms were excluded from analyses. As predicted, correct responses were faster for translation trials (mean: 1019 ms) than unrelated trials (mean: 1115 ms), *F*(1,17) = 22.70, *p* < 0.001, $\eta_{p}^{2}$ = 0.57 (see **Figure 2**). However, accuracy was slightly higher for unrelated trials (mean: 98%) than for translation trials (mean: 92%), *F*(1,17) = 38.86, *p* < 0.001, $\eta_{p}^{2}$ = 0.70, potentially indicative of a speed-accuracy trade-off.

**FIGURE 3:**
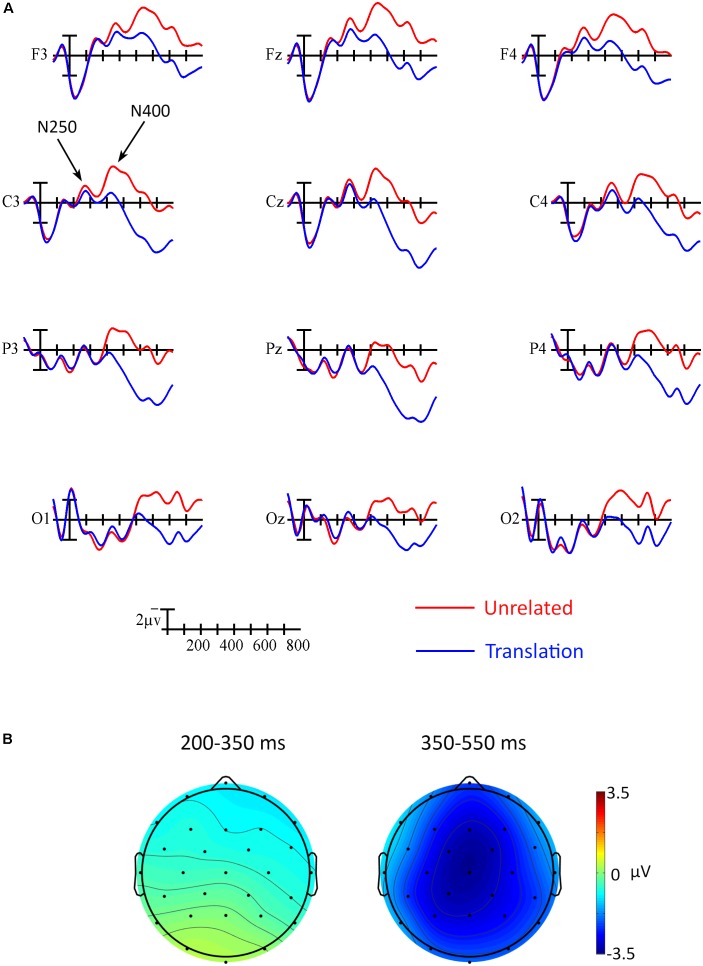
ERP results. **(A)** Grand averaged ERP waveforms elicited by targets in translation pairs (blue) and unrelated pairs (red). Each vertical tick marks 100 ms, the calibration bar marks 2 μV, and negative is plotted up. The 250 and N400 are indicated at site C3. **(B)** Scalp voltage maps showing the effect of translation priming (unrelated-translation) for each of the analyzed time windows.

**FIGURE 4:**
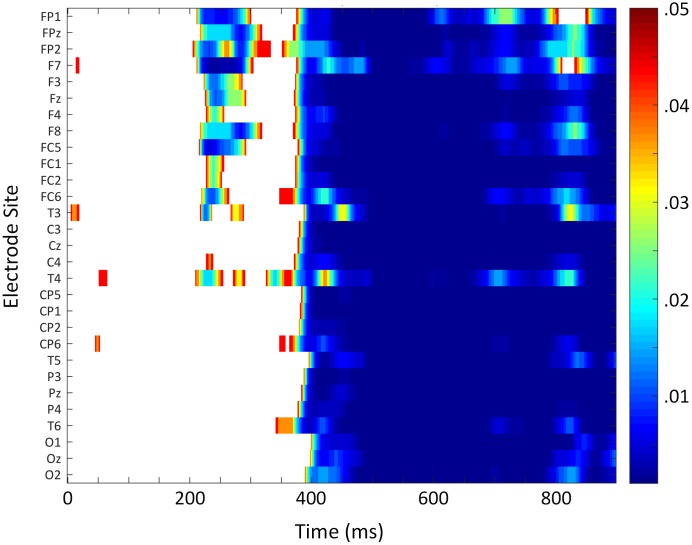
Time course analysis of ERP translation priming effect. False discovery rate-corrected *p*-values for each time point at each electrode site. Color indicates where the priming translation effect was significant.

In the ERP analyses, the main effect of Prime was not significant between 200 and 350 ms, *F*(1,17) = 0.88, *p* = 0.361, $\eta_{p}^{2}$ = 0.05. However, an interaction between Prime and Anterior/Posterior indicated that the effect went in the expected direction across anterior sites and in the opposite direction across the most posterior electrodes, Prime × Anterior/Posterior, *F*(3,51) = 5.73, *p* = 0.021, $\eta_{p}^{2}$ = 0.25 (see **Figure 3**). Follow-up analyses including only the most anterior electrodes (F3, Fz, and F4) confirmed that the priming effect was reliable at those sites, Prime, *F*(1,17) = 5.09, *p* = 0.038, $\eta_{p}^{2}$ = 0.23. A point-by-point time course analysis (see **Figure 4**) was consistent in suggesting that there was a weak early effect across frontal sites, but that the most reliable effect began within the N400 window, around 400 ms. Indeed, there was a widespread effect of priming within the N400 window (350–550 ms) that was strongest at central midline sites, Prime, *F*(1,17) = 26.88, *p* < 0.001, $\eta_{p}^{2}$ = 0.61, Prime × Laterality, *F*(2,34) = 4.34, *p* = 0.029, $\eta_{p}^{2}$ = 0.20, Prime × Anterior/Posterior, *F*(3,51) = 4.13, *p* = 0.043, $\eta_{p}^{2}$ = 0.19, Prime × Laterality × Anterior/Posterior, *F*(6,102) = 4.51, *p* = 0.004, $\eta_{p}^{2}$ = 0.21 (refer to **Figures 3, 4**).

## Discussion

Leading models of sequential bilingualism, including the RHM and BIA-d, posit that L2 words are processed via their L1 translation equivalents (i.e., lexical mediation) at low levels of proficiency. In contrast, there is growing empirical evidence to suggest that L2 words might be processed directly for meaning at relatively early stages of proficiency. To address this debate, we taught participants novel L2 words and tested them in a backward (L2–L1) priming paradigm with a short (140 ms) SOA. L1 targets in translation pairs elicited faster responses than the same targets in unrelated pairs, indicating that participants had learned the words and were processing them efficiently. ERP effects began as early as 200 ms after target onset at anterior sites, in a window that roughly corresponds to the N250. Such an early effect would appear to be consistent with lexical mediation and pre-activation of the lexical form of the L1 translation equivalent in these learners. However, the bulk of the observed ERP priming effects occurred within the N400 window, which suggests that L2 words were also directly activating their meanings. Given the short SOA, these results suggest that both L1 form and meaning representations were automatically accessed, but to different degrees. With a focus on the relative strength of the priming effects in the two windows, we discuss several potential lexical architectures that could underlie these results.

The early priming effects that we observed differ from typical N250 priming effects, which begin earlier and have a broader distribution. However, there are previous reports of an anterior N250 effect that more closely resembles the one we observed here. In particular, Grainger et al. (2006) found that orthographic overlap between visually-presented primes and targets modulates a posterior N250 whereas phonological overlap modulates a more anterior N250. In light of those results, one potential explanation for the anterior distribution of the early priming effect here is that (only) the phonological forms of the L1 words were primed. This makes sense given that participants learned the L2 words with pictures that they could name in their L1, but they never saw the orthographic forms of the L1 translation equivalents (until the ERP translation priming paradigm). If this interpretation is correct, it follows that when the orthographic forms of the L1 translations are presented during learning, the distribution of the early priming effects should include a more posterior component. Indeed, after teaching L2 words through lexical association, Pu et al. (2016) found early translation priming effects that appear to have a broader distribution than the effects that we observed here. Directly comparing the early priming effects for L2 words learned with L1 translations versus pictures in future studies would confirm that learning method influences the nature of the L1 form representations that are activated by L2 words.

In contrast, in a translation recognition paradigm with a 300 ms SOA, Ma et al. (2017) did not find a significant effect of form distractors within their P200 window (150–300 ms) and concluded that L1 translations are not automatically activated in low proficiency bilinguals. Several differences between the two studies could explain these divergent results. For one, our participants mastered a small set of L2 words in the context of this study whereas the participants in the study by Ma et al. (2017) were classroom learners and were therefore exposed to a wider range of L2 words in a variety of learning situations. How they learned the words may have affected the strength of activation of L1 translation equivalents. There were also methodological differences between the two studies that could help explain the results. For example, the translation recognition task that Ma et al. (2017) used only indirectly indexes activation of the translation equivalent; responses are recorded to neighbors of the L1 translations (i.e., form distractors) rather than to the L1 translations themselves. Therefore, it is possible that the L1 translation equivalents were also activated in that study, but not enough to interfere with processing of the neighbors. This seems especially plausible since the form priming effects that we observed were on the smaller side. The different SOAs between the two studies also likely influenced the results. It could be that activation of the L1 translation equivalent is transient such that it was strong enough to be measured at the 140 ms SOA here, but did not persist through the 300 ms SOA in the study by Ma et al. (2017). Evidence from priming studies with monolinguals supports this hypothesis; N250 (but not N400) effects become refractory at SOAs of 300 ms or more (Holcomb and Grainger, 2007). It is also important to note with a 140 ms SOA, the N250 window that we measured (200–350 ms after target onset) is temporally congruent with the N400 elicited by the primes (340–490 ms after prime onset). Some portion of this effect could therefore be driven by backward semantic priming from the L1 target to the L2 prime. More research is needed to test the effect of SOA in translation priming studies and, more generally, to determine which of these design differences led to the early priming effect here.

Although we found evidence of lexical mediation, the relative difference in size of the early lexical effect and the later N400 effect suggests that direct semantic activation was likely producing much of the priming effect. The typical centro-posterior distribution of our N400 effect suggests that it resulted from spreading of activation within the semantic system. This contrasts with the fronto-central N400 priming effect that Mestres-Missé et al. (2007) found in a 500 ms SOA backward priming paradigm with learners. In that study, participants implicitly learned the meanings of novel words presented at the end of three L1 sentences with increasing contextual constraint and were tested in the priming paradigm the same day. The authors attributed the frontal distribution of the priming effect to recruitment of prefrontal regions and an increase in cognitive control during semantic retrieval of the new words. These two studies might represent two different stages of L2 learning as described, for example, in the episodic hypothesis of L2 learning (e.g., Jiang, 2000; Jiang and Forster, 2001; Witzel and Forster, 2012). Proponents of the episodic hypothesis differentiate between “lexical knowledge,” which involves storing information about L2 words in general episodic memory and “lexical competence,” which denotes that lexicosemantic information has been integrated into the linguistic system. The frontal N400 effect reported by Mestres-Missé et al. (2007) could be indicative of the controlled meaning retrieval that characterizes the lexical knowledge stage, whereas the more typical N400 distribution that we observed in the present study suggests that L2 words can be integrated into the lexicosemantic system over a span of only a few days.

How do we account for activation of both the L1 translation equivalent and the meaning? It would appear that these data reflect a combination of lexical mediation and semantic mediation, or the transition from one to the other. In both the RHM and the BIA-d, the lexical links decrease in strength as proficiency increases and direct semantic links are established, but they never disappear entirely. Thus, it is possible that these words were being primarily processed through the semantic route, but residual activation was also flowing to the L1 translation equivalent via the weakened lexical links. It could also be that individual L2 words were at different stages of the transition from lexical mediation to semantic mediation (see, e.g., Kroll and Tokowicz, 2005). In other words, the two patterns in the averaged ERPs might reflect processing via lexical mediation for a (small) subset of the L2 words and processing via semantic mediation for a (larger) subset of the L2 words. In the BIA-d, this transition is implemented by decreasing the lexical “clamping” between each L2 word and its translation equivalent and increasing top–down inhibition of the L1 from the L2 language node (Grainger et al., 2010). We know from studies with proficient bilinguals that the translation priming effect should onset within the N400 window in the final state (e.g., Phillips et al., 2006; Duñabeitia et al., 2010b; Geyer et al., 2011). This could be achieved either by further weakening of the lexical links for all words or by processing a larger majority of the L2 words via semantic mediation. If the latter is true, and the transition is happening at the level of individual words, it would be informative to know what lexicosemantic characteristics allow certain words to transition faster than others.

## Conclusion

The present study offers new evidence for both early (N250-like) and later (N400) translation priming effects at a short SOA that precludes strategic processing. The N400 priming effect was substantially larger than the earlier anterior effect. It is therefore unlikely that it resulted purely from the indirect (i.e., lexically mediated) semantic processing posited in the RHM and BIA-d. Rather, the data are more consistent with direct semantic access after relatively few exposures to new L2 words. Whether all of the L2 words were being processed via this direct semantic pathway is not clear. The early form priming effects could be due to weak activation of the L1 translation equivalents of all L2 words or, alternatively, to strong activation of the L1 translation equivalents of a small subset of L2 words that were still being processed via lexical mediation. How these dynamics would differ among classroom students who learn a more diverse set of words as part of a more ecologically valid language learning experience also remains unknown. Tracking the relative contributions of lexical versus semantic mediation over the course of learning, including in adults who learn their L2 in more typical classroom settings, will begin to answer these important questions.

## Ethics Statement

All participants gave written informed consent in accordance with the Declaration of Helsinki. The protocol was approved by the Institutional Review Board at San Diego State University.

## Author Contributions

GM and PH designed the study. GM collected and analyzed the data. GM, KM, and PH participated in writing the paper.

## Conflict of Interest Statement

The authors declare that the research was conducted in the absence of any commercial or financial relationships that could be construed as a potential conflict of interest.

## Footnotes

1 Whether P200 or N250 amplitude is reported seems to depend largely on the task that was used. The P200 is often reported in translation recognition studies in which the form-related distractor is predicted to be more difficult to process. In contrast, the N250 is more often reported in translation and identity priming studies in which the related conditions are predicted to be easier to process.

2 Note that analyzing the data from all 20 participants who were included the original report yields the same pattern of results that we report here.
